# Early Amyloidogenic Oligomerization Studied through Fluorescence Lifetime Correlation Spectroscopy

**DOI:** 10.3390/ijms13089400

**Published:** 2012-07-25

**Authors:** Jose M. Paredes, Salvador Casares, Maria J. Ruedas-Rama, Elena Fernandez, Fabio Castello, Lorena Varela, Angel Orte

**Affiliations:** 1 Department of Physical Chemistry, Faculty of Pharmacy, Campus Cartuja, Granada, 18071, Spain; E-Mails: paredes@correo.ugr.es (J.M.P.); mjruedas@ugr.es (M.J.R.-R.); fabiocastello@ugr.es (F.C.); 2 Department of Physical Chemistry, Faculty of Sciences, Campus Fuentenueva, Granada, 18071, Spain; E-Mails: scasares@ugr.es (S.C.); fdez@ugr.es (E.F.); lvarela@ugr.es (L.V.A.)

**Keywords:** amyloids, protein aggregation, pulsed interleaved excitation, protein oligomers, single-molecule fluorescence

## Abstract

Amyloidogenic protein aggregation is a persistent biomedical problem. Despite active research in disease-related aggregation, the need for multidisciplinary approaches to the problem is evident. Recent advances in single-molecule fluorescence spectroscopy are valuable for examining heterogenic biomolecular systems. In this work, we have explored the initial stages of amyloidogenic aggregation by employing fluorescence lifetime correlation spectroscopy (FLCS), an advanced modification of conventional fluorescence correlation spectroscopy (FCS) that utilizes time-resolved information. FLCS provides size distributions and kinetics for the oligomer growth of the SH3 domain of α-spectrin, whose N47A mutant forms amyloid fibrils at pH 3.2 and 37 °C in the presence of salt. The combination of FCS with additional fluorescence lifetime information provides an exciting approach to focus on the initial aggregation stages, allowing a better understanding of the fibrillization process, by providing multidimensional information, valuable in combination with other conventional methodologies.

## 1. Introduction

The amyloid fibril formation mechanism is a topic of intensive research given its relationship to a wide variety of human diseases that have high economic and social impacts, some of which are severely impairing, such as Alzheimer’s and Parkinson’s diseases. There is currently no effective therapy or active prevention policy for these diseases due to a lack of understanding of their molecular mechanisms and the factors that trigger them. The formation of amyloid fibrils occurs through the formation of initial aggregates (lag or nucleation phase) that exponentially grow into mature protofibrils, and finally mature fibrils (elongation phase), following a scheme similar to that of crystallization [1,2]. The sigmoidal growth profile reflects a preference for addition of the monomers onto existing aggregates via seeding rather than over the higher energy barrier to form new oligomers [3]. However, additional secondary processes such as fibril fragmentation result in increasingly complex kinetics [4]. This mechanism has been observed for all proteins involved in amyloidogenic diseases. Furthermore, some proteins unrelated to disease, such as the SH3 domain of the phosphatidyl-inositol 3-kinase, can undergo these processes under certain conditions [5]. Therefore, this amyloidogenic aggregation pathway appears to be common to all proteins and peptides under certain environmental conditions [6,7]. We have observed that a single mutation located at the distal loop of the α-spectrin SH3 domain (N47A Spc-SH3) favors a fast conversion of the protein into amyloid fibrils under mildly acidic conditions [8]. This result allowed us to use this small globular domain as a model to investigate the protein conformational changes that trigger aggregation. Recently, we also observed that the difference in the fibrillation rate of this variant of the domain with respect to the wild-type form and some other mutants relies on an enhancement in the rate of appearance of transient oligomers favored by this specific mutation, which increases the nucleation rate without an overall destabilization of the native state [9]. In addition, the Spc-SH3 fibrillation process is strongly accelerated by temperature and the presence of salts, and it is only slightly enhanced by acidification [10]. Over the last two decades, significant effort has been made to identify, isolate and characterize, both structurally and functionally, the oligomeric species that appear during the initial stages of amyloidogenesis. These studies are important in light of the increasing evidence indicating that these species are responsible for the cytotoxicity and, therefore, the etiology of these disorders [6,11–15].

Due to their importance, the initial oligomers formed during fibril formation should be studied in detail. The population of early aggregates and the rates at which they develop is inherently heterogeneous and transient; therefore, the techniques employed for their study must overcome this molecular particularity. Recently, a family of techniques has been developed that is capable of observing individual molecules in solution and in real time. Application of these techniques to biophysical and biomedical systems is revolutionizing the way that scientists think of biomolecular systems [16–18]. The use of fluorescent tags to label the studied protein under tightly focused laser light, using a confocal microscope and extremely sensitive detection, allows the direct probing of individual proteins. In addition, their progress can be followed during aggregation. Based on the variation in the brightness of the fluorescence events, growing species can be sorted according to size [19]. If the fluorescence signals are time-correlated with themselves, so-called fluorescence correlation spectroscopy (FCS), the output curve provides information on the diffusion velocity of the labeled species and can be used to follow the oligomer growth and size distributions [20,21]. More advanced techniques utilize two coaggregating populations of the studied protein, labeled with two different dyes and two different-colored corresponding excitation lasers. By observing coincident single-molecule fluorescence events, the formation of oligomers can be identified and analyzed [22–24]. Oligomer size distributions and their time variation have been obtained using this two-color coincidence detection (TCCD) method for aggregating SH3 domains [23], neuroserpins [24], amyloid-β [25], or α-synuclein [26]. The excitation laser light is a continuous wave; therefore, the emitted fluorescence intensity is the only detection parameter. However, the fluorescence emission is intrinsically a multiparametric process because other parameters can be accessible, such as the fluorescence lifetime, anisotropy, spectral dispersion, and so forth. Thus, the single-molecule fluorescence methodologies can benefit from widening the dimension of the detected information. For instance, single-molecule multiparameter fluorescence detection [27] can be achieved by sorting the single-molecule events according to their fluorescence intensity, lifetime, and anisotropy. This realization requires new technical and methodological advancements that enhance the application of the single-molecule techniques in biophysics. One of these advances is the merger of single-molecule fluorescence spectroscopy with time-correlated single-photon counting techniques using pulsed lasers and appropriate single-photon detectors and electronics. This combination is feasible because of the time-tagged time-resolved (TTTR) data acquisition mode. TTTR allows discrimination of the photon arrival time with respect to the excitation pulse and, hence, reconstruction of the fluorescence decay traces from the entire measurement [28]. This merger has resulted in improvements such as time-gated FCS [29] and fluorescence lifetime correlation spectroscopy (FLCS) [30–32]. FLCS overcomes certain instrumental limitations inherent in conventional FCS by utilizing time-weighted filters from the time-resolved profile of the fluorophores. FLCS selectively resolves the diffusion times of spectrally overlapped fluorophores and yields accurate estimations of the molecular concentration in the excitation volume [31] and the dynamics of micellar membranes [33], vesicles [34], and DNA compaction [35]. Furthermore, lifetime information and temporal resolution can be integrated into a two-color configuration with an adequate pulsed laser and driver electronics. The best approach involves spatially overlapping the two lasers to obtain the information available for TCCD while delaying one laser with respect to the other. This procedure is the so-called pulsed interleaved excitation (PIE) through which the arriving photons are sorted according to the excitation laser pulse [36]. Improved crosstalk removal, which accounts for the quenching effects, and perfected energy transfer estimation are advantages offered by PIE.

We have exploited the technical advantages of advanced single-molecule fluorescence two-color PIE to perform FLCS and applied it to study the growth and kinetics of oligomers formed during the incubation of the N47A Spc-SH3 domain. This work demonstrates how these developments in single-molecule techniques can ultimately solve relevant biophysical problems.

## 2. Results and Discussion

### 2.1. Translational Diffusion Coefficients Show Oligomer Growth Kinetics

**Figure 1:**
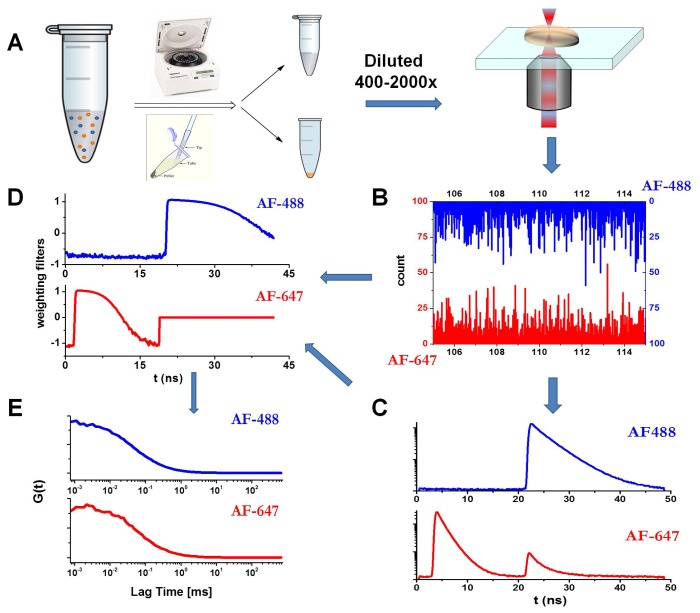
Schematics of the Pulsed Interleaved Excitation-Fluorescence Lifetime Correlation Spectroscopy (PIE-FLCS) measurement. (**A**) Protocol for the PIE-FLCS measurements. (**B**) Example of fluorescence traces in the AF488 and AF647 channels. (**C**) TTTR methodology allows independent reconstruction and selection of fluorescence decay traces of AF488- (detector 1) and AF647-labeled (detector 2) proteins. (**D**) Weighting filters for correlation. (**E**) Lifetime-filtered autocorrelation curves in both channels.

We used FLCS, in combination with a PIE excitation scheme, to analyze the autocorrelation curves of the soluble fraction of the incubated aliquots of the N47A Spc-SH3 domain to follow the early aggregation kinetics and to extract size distributions. For this, the N47A Spc-SH3 domain was labeled with either a green (Alexa Fluor 488, AF488) or a red fluorophore (Alexa Fluor 647, AF647). The incubation in amyloidogenic conditions was performed with an approximate labeled-to-unlabeled protein ratio of 1:700 (see the Experimental section). The aliquots collected during incubation were centrifuged, as detailed in the Experimental section. We kept and analyzed only the soluble fraction that remained in solution. This step allowed us to focus on the small, early amyloidogenic aggregates. The soluble fraction of the oligomers was diluted on a microscope slide to collect fluorescence emission traces. The dilution factors were those that maintained a high concentration of unlabeled protein and a concentration of labeled protein in the pM range (the exact concentration cannot be known due to the separation of the soluble and insoluble fractions). Figure 1 presents the measurement schematics and details: an example of the fluorescence decay traces in the AF488 and AF647 channels (Figure 1B), the collected overall decay traces in both channels (Figure 1C), and the corresponding estimated weighting filters (Figure 1D) to determine the lifetime filtered autocorrelation curves. The application of FLCS allows a more accurate determination of the dye concentrations by avoiding the afterpulse of the detector and removing the background contribution [31]. We focused on both the AF488 filtered autocorrelation curve with direct excitation at 470 nm and the AF647 filtered autocorrelation curve with direct excitation at 632 nm. The PIE methodology allows the application of appropriate time windows to select photons that arrived at either detector just after the first or the second laser excitation pulse (Figure 1C). Figure 1E presents the corresponding filtered autocorrelation curves.

First, we examined the average diffusion coefficient of the N47A Spc-SH3 aggregates from the FLCS curves. To examine the diffusion coefficient, accurate calibration of the detection volume was required. A reference dye, Tokyo Green II (TG-II), with a diffusion coefficient of 371 μm^2^·s^−1^ at 20 °C was used for this purpose [37]. We obtained the FLCS curve from TG-II and, by fitting it to the diffusional model (Equation 1), the probe volume, *V_probe, AF488_*, of the green channel was determined to be 0.76 fL. The next step was to obtain the diffusion coefficient of the monomeric protein. For this step, we recorded the fluorescence emission traces from the non-incubated, AF488-labeled N47A Spc-SH3, applied the protocol to obtain the FLCS curve, and fit the curve to the general FCS equation (Equation 2). To ensure reproducibility, we repeated the entire process with three different preparations of labeled, monomeric N47A Spc-SH3. The average diffusion coefficient of the monomeric N47A Spc-SH3 was determined to be 250 ± 14 μm^2^·s^−1^. With this diffusion coefficient, the calibration of the 633-nm probe volume was performed by analyzing the FLCS curves from the non-incubated, AF647-labeled N47A Spc-SH3. We observed that the red channel probe volume was *V_probe, AF647_* = 1.68 fL. The obtained diffusion coefficient for the N47A Spc-SH3 domain is larger than the theoretical value based on the Stokes-Einstein equation, calculated using the HYDROPRO v10 software [38] for hydrodynamic properties, which was 170 μm^2^·s^−1^. This difference may arise from the effect of the dyes (not accounted in the calculations), the effect of pH and other buffer species over the structure of the protein, or small changes in the temperature. However, our experimental result is robust and reproducible. Therefore, we established the value 250 μm^2^·s^−1^ as the correct one for the N47A Spc-SH3 monomer.

**Figure 2:**
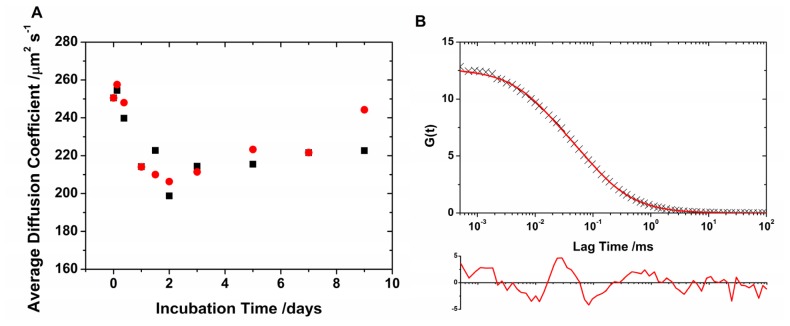
(**A**) Average diffusion coefficient as a function of incubation time for AF488- (black) and AF647-labeled (red) N47A Spc-SH3. (**B**) Example of lifetime-weighted autocorrelation curve (AF488 channel, 48 h of incubation time) fitted using a single diffusive species (Equation 2). The residuals plot (below) indicates a poor fit.

Once the calibration of the two overlapping probe volumes was established, the average diffusion coefficients of the different aliquots collected during the N47A Spc-SH3 incubation were recovered. The fluorescence emission traces were analyzed to simultaneously obtain lifetime-filtered autocorrelation curves for both channels, AF488 and AF647. The FLCS curves were fitted to Equation 2 to obtain the average diffusion times, τ_D_, and the average diffusion coefficients using Equation 3 (Figure 2A). However, these fits, which used a single diffusive species, are not particularly accurate judging by the correlation in the residuals and the high values of χ^2^ (Figure 2B). The slowed diffusion reveals the aggregation of the monomeric N47A Spc-SH3 into higher ordered units, and the poor fit indicates that the actual population cannot be described with a single diffusive species. The shift in the diffusional region of the autocorrelation curves provides evidence for the formation and growth of slowly diffusing oligomers and the effective incorporation of the labeled monomers into such oligomers. However, the oligomer population consists of a heterogeneous mixture. Note that the cross-correlation of the fluorescence traces in the AF488 and AF647 channels does not exhibit any amplitude because of the small concentration of labeled monomers as compared with the unlabeled units. We also examined the population of oligomers by analyzing the FLCS curves in detail as described in the next section.

### 2.2. Oligomer Size Distributions from the FLCS Curves

**Figure 3:**
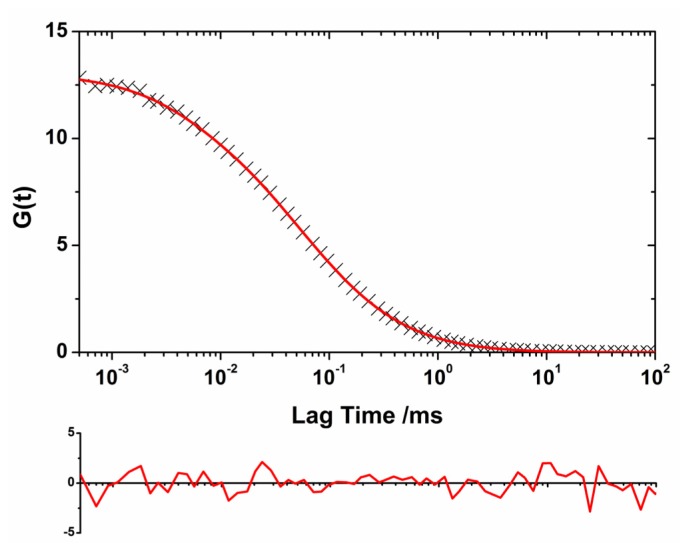
Example of lifetime-weighted autocorrelation curve (AF488 channel, 48 h of incubation time) fitted using Equation 7, the convolution of a log-normal distribution of sizes with the general autocorrelation function. The residuals plot (below) indicates the accuracy of the fit.

**Figure 4:**
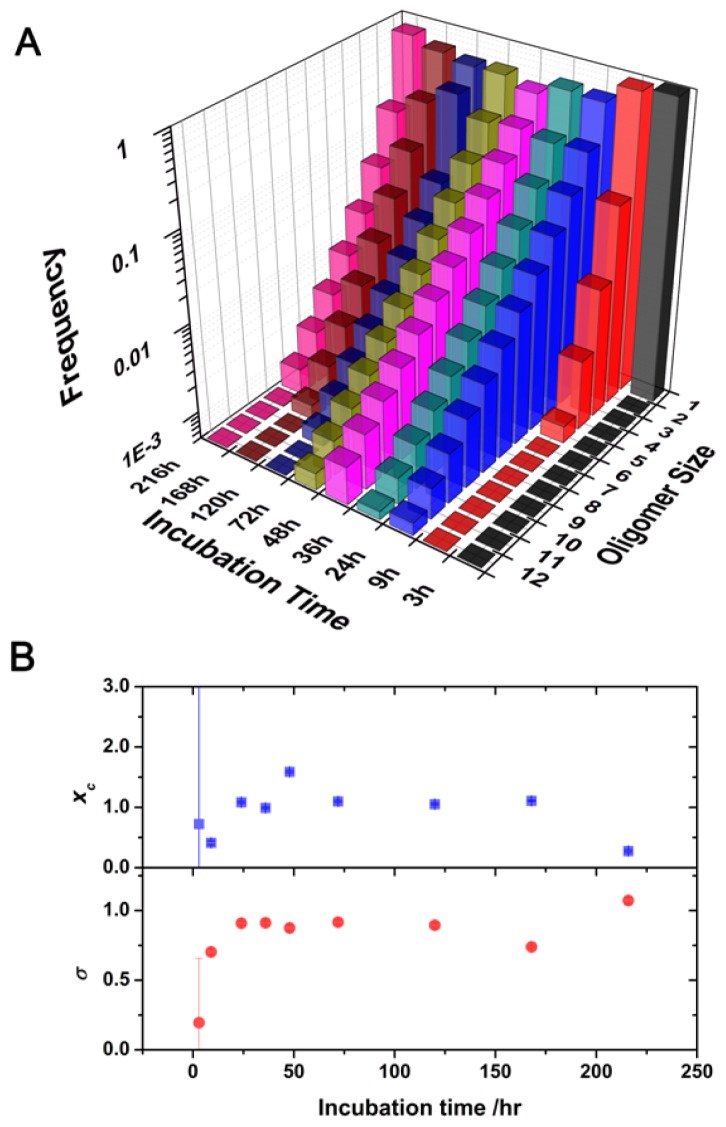
(**A**) Oligomer size distributions as a function of incubation time. (**B**) Plot of the fitting parameters *x_c_* (top) and *σ* (bottom) of the log-normal distributions in Figure 4A.

**Figure 5:**
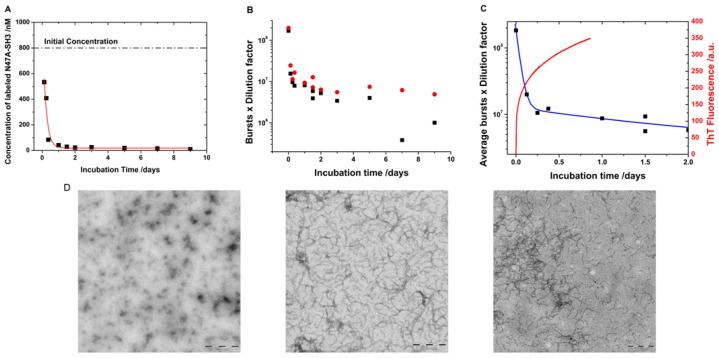
(**A**) Concentration of labeled monomers obtained from the fitted lifetime-weighted autocorrelation functions. The line represents exponential decay fits. (**B**) Single-molecule fluorescence bursts, corrected using the different dilution factors from the AF488 (black) and AF647 (red) channels. (**C**) Average single-molecule fluorescence bursts, corrected by the different dilution factors, (black symbols) fitted to a double exponential decay function (blue line). The figure also presents the corresponding Thioflavin T (ThT) fluorescence emission trace (red line) of an unlabeled sample incubated under the same experimental conditions. (**D**) Transmission electron micrographs of the incubated samples after 0 h (left), 4 h (middle), and 48 h (right) of incubation. Scale bars represent 200 nm.

A heterogeneous population of oligomers of different sizes exhibits complex autocorrelation functions. We developed a model to extract the size distribution of the oligomers present in the soluble fraction of the aliquots by convoluting a log-normal distribution of sizes with the autocorrelation function as detailed in the Experimental section. We chose a log-normal distribution because experimental [23,25] and theoretical evidence [39,40] indicated that this distribution is the most probable for the oligomer sizes. We used this methodology to obtain the size distribution of the soluble oligomers as the incubation proceeded. The use of two different reporter dyes, AF488 and AF647, and the capabilities of the dual-color interleaved excitation allowed the simultaneous generation of two independent lifetime-filtered autocorrelation curves for each time-point, as previously described. Therefore, we obtained two independent estimations of the size distribution from a single experiment. The fit performed using this method presented both a decrease in the χ^2^ value of at least 15% and a more random distribution of the residuals as compared with the fit using the single diffusive species function (Figure 3). Figure 4A shows the size distribution of the SH3 Spc-N47A soluble oligomers at various incubation times, which were averaged from the results for the two different dyes and different repetitions. Figure 4B shows the fitting results for the central positions and widths of the log-normal distributions. In the initial aliquots, the distributions were narrow and sharp, corresponding primarily to monomeric proteins and small quantities of early aggregates, especially in the first datapoint causing a large associated error. As the incubation proceeded, the size distributions shifted toward larger sizes, indicating oligomer growth and an increase in the heterogeneity of the oligomer population. The size distribution of the soluble oligomers peaked at 48 h of incubation; then, it appeared to shrink to smaller values. This effect is primarily caused by the further growth of large aggregates that entered the insoluble fraction during centrifugation. A similar trend has been previously observed in the aggregation of a different SH3 domain using single-molecule fluorescence [23]. Remarkably, a detectable population of soluble oligomers remained in solution after five days of incubation, even though the sample was primarily fibrillar as indicated by electron micrographs [8] (see Figure 5D). This result can be interpreted as indicating a population of oligomers whose incorporation into mature fibrils is ineffective due to an inadequate oligomer structure. It has been frequently hypothesized that amyloidogenic oligomers must undergo a structural rearrangement before their incorporation into mature fibrils to match the appropriate β-sheet-rich conformation. Previous experimental evidence has demonstrated this phenomenon [21,23], including a recent report by Cremades *et al.* in which oligomers of α-synuclein (the protein linked to Parkinson’s disease) with different structural arrangements present different cytotoxicities [26]. In contrast, our result may be related to the dynamic nature of the aggregation process, by which the mature fibrils can be a constant source of small oligomers [4] as demonstrated in recent experiments on fibril disaggregation of amyloid-β [25] and α-synuclein [26]. For our experiments with N47A Spc-SH3, the precise role of the residual oligomers remains uncertain, and further work on this subject is currently being undertaken.

Our methodology provides size distributions, in a similar manner than TCCD can [23], however focusing on the diffusional properties of the oligomers. This methodology only needs a small fraction of labeled protein, thus, minimally altering the behavior of the native, unlabeled protein. This knowledge on the early aggregates could be completed for the late deposits by using fluorescence microscopy techniques, especially taking advantage of substances that specifically bind amyloidogenic material and change their fluorescent properties upon binding, such as *de novo* designed luminescent conjugated polymers [41,42]. Other microscopy techniques, such as fluorescence anisotropy microscopy [43] and fluorescence lifetime imaging [44], can report valuable data on the mature aggregates. Nevertheless, the single molecule fluorescence methodologies are definitively more powerful towards the initial, small oligomers. For instance, our FLCS-based methodology takes advantage of the multidimensional nature of the measurements to extract kinetic information, as it will be discussed in the following section.

### 2.3. Kinetics of Monomer Incorporation

In addition to the size distributions, the single-molecule fluorescence measurements provide kinetic information on the aggregation of the N47A Spc-SH3 domain. The parameter *N* from the fits of the diffusional portion of the autocorrelation function (Equation 1) represents the average number of labeled molecules in the probe volume during the collection of the fluorescence traces. Therefore, because the volume of the laser probe is calibrated (*V_probe_*), the concentration of labeled molecules in the microscope coverslip can be obtained as *N*/*V_probe_*. Our dual-color, interleaved excitation approach allows the simultaneous determination of the concentration of both the AF488- and AF647-labeled monomers. To obtain an accurate, comparable determination of the concentration in the initial aliquot, prior to dilution on the coverslip, the dilution factors must be considered. Figure 5A presents the average concentration of the labeled monomers in the soluble fraction as a function of the incubation time. During incubation, the collected aliquots were separated into two fractions, soluble and insoluble (Figure 1A). As the amyloid fibril formation moved forward, more protein was added to form larger aggregates, and protofibrils remained in the insoluble fraction. Therefore, the overall concentration of the protein and that of the labeled protein in the soluble fraction decreased with increasing incubation time. This loss of protein in the soluble fraction was propagated to our single-molecule measurements, resulting in the trend shown in Figure 5A. To extract kinetic information regarding the velocity of monomer incorporation into the insoluble fraction, we fitted the data in Figure 5A to an exponential decay function (*y* = *A* × e^−^*^k^*^·^*^t^* + *y*_0_ in which *k* is the apparent rate of monomer loss). The fit in Figure 5A provides a monomer loss rate of 0.21 ± 0.05 h^−1^, which corresponds to a decay time (apparent lifetime of monomer = 1/*k*) of 4.9 ± 1.2 h.

In addition, a second, independent method can be used to study the same kinetic process embedded within the same experimental measurements. The fluorescence traces in both channels (Figure 1B) were collected under single-molecule regime in which individual, non-overlapped fluorescent bursts can be counted in each channel. The single-molecule burst rate is proportional to the concentration of the labeled species [22]. Consequently, fluorescence burst counting is an excellent tool for following changes in the concentration over time. In fact, the fluorescence burst rates in both the AF488 and AF647 channels indicated a clear decrease with incubation time (Figure 5B) following correction using the different dilution factors employed in the measurements. This result indicates that, as the aggregation proceeded, more protein (and thus, more labeled protein) was incorporated into the insoluble fraction of the aliquots in which the largest aggregates and fibrils were collected. The experimental burst rates are, however, better fitted using a double exponential decay function to obtain an independent estimation of the apparent monomer incorporation rate (Figure 5C). A value of 0.97 ± 0.08 h was obtained as an average value between the AF488 and AF647 channels for the rapid decay component. A second, slow component of 42 ± 17 h represents the slower monomer loss at later incubation times given the low free-monomer concentration.

These aggregation kinetics can be compared with conventional bulk measurements such as the fluorescence measurements of Thioflavin T (ThT), a specific dye reporter of β-sheet structure. ThT fluorescence obtained from an unlabeled, incubated N47A Spc-SH3 sample, under the same incubation conditions, exhibited rapid growth (Figure 5C). The ThT fluorescence trace can be fitted to a double exponential growth function (*y* = −*A1* × e^−^*^k1^*^·^*^t^* − *A2* × e^−^*^k2^*^·^*^t^* + *y*_0_) resulting in two kinetic growth phases. The first growth time (1/*k1*) was 0.42 ± 0.01 h, and the second growth time was 10.7 ± 0.1 h; both times are in good agreement with the decays phases obtained in the single-molecule experiments. This fast kinetics is in agreement with the transmission electron micrographs that show clear evidence of extensive fibrillation yet at early incubation times around 4 h (Figure 5D). Note that the kinetic figures reported here are valid for the specific incubation conditions probed and only hint at the velocity of the reaction. The aggregation kinetics depends on the overall protein concentration and other effects such as pH, temperature and salt concentration [8–10]. More thorough kinetic models [3,4] can be tested by studying different experimental conditions and their effects. As a direct comparison, the monomer aggregation kinetics reported herein for N47A Spc-SH3 are in good agreement with those observed for another SH3 domain that also formed amyloid fibrils at pH 3.2 and exhibited a monomer incorporation decay time of 3.1 h [23].

The ensemble fluorescence techniques, like those measuring ThT or congo red binding, are broadly employed and deliver interesting results on amyloid formation kinetics [45]. There exist other small binding molecules that change their emission properties upon binding to pre-fibrillar material, such as 4-(dicyanovinyl)-julolidine and derivatives of 1-amino-8-naphthalene sulfonate [46,47]. The ensemble steady-state fluorescence measurement provides a way to follow the kinetics of those species to which the active fluorophores are bound. However, different dyes may show dissimilar binding affinities probing various states of the amyloidogenic process [48], and hence, not showing specificity toward certain types of oligomers. More advanced ensemble fluorescence techniques can be employed. For instance, steady-state and time-resolved anisotropy give an idea of the rotational diffusion properties of the oligomers, which is slowed down upon oligomer growth [48,49]. Fluorescence anisotropy measurements are, however, limited by the fluorophore’s fluorescence lifetime. Long-lived fluorophores, with lifetimes around 200 ns, have been shown to provide higher resolution for larger aggregates [50]. In any case, all these bulk techniques always suffer from the same inconvenient: By probing a large population of molecules, only average values are recovered, and detailed insights into the population of oligomers are hidden in the ensemble averaging [17]. Our technique, as well as other single-molecule fluorescence-based methodologies to study amyloid aggregates [20,21,23,51], has the capability of providing kinetic information, of similar quality than ensemble, conventional methodologies, but also give the opportunity to take a closer look into the oligomeric population.

## 3. Experimental Section

### 3.1. Expression of the N47A Spc-SH3 Domain

The SH3 domain of α-spectrin does not contain cysteine residues in its primary sequence. However, cysteine is required to produce the fluorophore-tagged derivatives needed for these studies, so a new variant of this domain containing a six-residue tag (Gly-Ser-Gly-Ser-Gly-Cys) at the *C*-terminus was engineered from the N47A mutant of the domain. The engineered mutant ending with the free cysteine (N47A-Cys) was prepared from the pET3d plasmid containing the modified domain’s clone (ordered from GeneArt^®^ Life Technologies GmbH, Germany). The protein was overexpressed from that plasmid in a BL21 (DE3) strain of *E. coli* cells. The collected cells were lysed via sonication in a 5 mM sodium citrate buffer at pH 3.0. Because the protein is highly soluble under mildly acidic conditions, the lysate was acidified to a pH value of approximately 3.0. The protein was then recovered from the supernatant by precipitation with ammonium sulfate at 75% saturation. The precipitated N47A-Cys protein was solubilized in 20 mM sodium phosphate buffer, 100 mM NaCl, pH 7.0, containing 7 M urea. The protein was finally purified by size exclusion chromatography on a HiLoad Superdex 75 column (GE Healthcare Life Sciences Corp., USA) and extensively dyalized to remove urea in 20 mM glycine buffer, pH 3.0, containing 5 mM dithiothreitol (DTT) to avoid crosslinking of the free cysteine residues. Protein purity was confirmed via SDS-PAGE.

### 3.2. Labeling and Purification of the Protein

The N47A-Cys SH3 domain was labeled with either maleimide-modified Alexa Fluor^®^ 488 (AF488) or Alexa Fluor^®^ 647 (AF647) dyes (Invitrogen, Carlsbad, CA, USA) via the cysteine thiol moiety in 50 mM phosphate buffer at pH 7.2 following the supplier specifications. A dye-to-protein ratio of 5:1 was used to enhance the dye reactivity. Prior to use, the labeled protein was purified from the excess free dye using Amicon^®^ Ultra Centricon filter units (Millipore, Billerica, MA, USA), dialyzed and lyophilized. The fluorescence properties of the fluorophores upon labeling were unaltered, as no appreciable quenching was detected in their fluorescence lifetime (see Figure 1C). The average fluorescence lifetimes of AF488 and AF647 within the protein were 3.8 ns and 1.6 ns, respectively. These lifetimes were very similar to those reported for the unquenched dyes of 4.1 and 1 ns (the AF647 lifetime is known to increase upon labeling) [52].

### 3.3. Incubation and Formation of Amyloid Fibrils

Unlabeled N47A Spc-SH3 samples at a concentration of 8.3 mg/mL (1.1 mM) were incubated at 37 °C in the presence of 0.8 μM N47A Spc-SH3 labeled with AF488 and AF647 in buffer (100 mM glycine, pH 3.2, 100 mM NaCl). The buffer was filtered through 0.02 μm filters (Whatman) prior to use. The samples were collected at different incubation intervals, between 0 and 9 days. Soluble (S) and fibril fractions (P) were isolated by ultracentrifugation for 3 h at 30,000 rpm at 4 °C. The samples were suitably diluted to obtain optimal conditions for the data collection. Aliquots were kept in darkness and frozen at −50 °C when not in use to avoid possible protein precipitation and dye deterioration. Measurements were performed in triplicate.

### 3.4. Thioflavin T Binding Assay

Thioflavin T (ThT) specifically binds to β-sheet structures and allows the monitoring of amyloid fibril aggregation [53]. We performed ThT fluorescence measurements using a Varian Cary Eclipse spectrofluorimeter (Agilent Technologies, Santa Clara, CA, USA) equipped with a Peltier-controlled thermostatic cell holder. The aggregation process was started by mixing a freshly prepared protein solution with a concentrated stock solution of the dye previously prepared in the same buffer (100 mM Gly, 100 mM NaCl, pH 3.2) to reach a final concentration of 10 μM ThT. Prior control experiments were used to assess the optimal final concentration of dye in the sample. The sample was placed into a fluorescence cuvette, previously thermostatized at 37 °C and covered with mineral oil to prevent evaporation. The fluorescence emission intensity of ThT was collected in real time at 485 nm using an excitation wavelength of 440 nm.

### 3.5. Transmission Electron Micrographs

Transmission electron micrographs of the incubated samples at different incubation times were collected by taking aliquots deposited on Formvar 300-mesh copper grids, washed twice with MilliQ water, and stained using 1% (*w*/*v*) uranyl acetate, and finally dried at 37 °C for 6 min. The microscope was a Libra 120 plus (Carl Zeiss SMT, Germany), operating at 120 kV, equipped with a LaB_6_ filament and a SSCCD 2k × 2k direct coupling camera.

### 3.6. Fluorescence Lifetime Correlation Spectroscopy (FLCS) with Pulsed Interleaved Excitation (PIE)

Fluorescence fluctuation traces were collected in a MicroTime 200 fluorescence lifetime microscope system (PicoQuant GmbH, Germany) using the time-tagged time-resolved (TTTR) [28] methodology, which enables a reconstruction of the decay histogram. The excitation sources were 470- and 633-nm LDH pulsed lasers with minimum pulse widths of 73 and 88 ps, respectively. The light beam was directed into the sample by an FITC/CY5 dichroic beamsplitter (AHF/Chroma) and a 1.4 NA, 100× oil immersion objective mounted on an Olympus IX71^®^ inverted microscope. The collected fluorescent light was filtered with a long-pass filter HP500LP filter (AHF/Chroma) and focused onto a 75-μm pinhole. After passing through the aperture, the transmitted light was separated by a 600dcxr dichroic beamsplitter (AHF/Chroma) into green (donor) and red (acceptor) fluorescence; then filtered with FF01-520/35 (Semrock) and HQ685/70m (AHF/Chroma) band-pass filters, respectively. Each beam was refocused into an avalanche photodiode detector (SPCM-AQR SPAD, PerkinElmer). The data acquisition was performed with a TimeHarp 200 TCSPC module (PicoQuant) in TTTR mode.

The fluorescence decay traces were analyzed using SymPhoTime software (PicoQuant), applying the maximum likelihood estimator (MLE), which yields correct parameter sets for low count rates [54]. Having recovered the time-resolved profiles, the weighting filters were calculated for FLCS using the decay traces obtained from high concentration samples. Once the temporal filters were established, the fluorescence signal was correlated. Without photophysical processes affecting the fluorescence fluctuations and assuming a prolate ellipsoidal Gaussian excitation probe volume, the autocorrelation function corresponds to the following equation [55]:

(1) $$G_{D}(t)=\frac{1}{N}{\left(1+\frac{t}{\tau_{D}}\right)}^{-1}{\left(1+\frac{t}{\tau_{D}\cdot a^{2}}\right)}^{-1/2}$$

in which *t* is the correlation lag time, *τ_D_* represents the diffusion time, *N* is the average number of molecules in the probe volume, and *a* is the ratio between the effective focal radius along the optical axis, *a_z_*, and the lateral focal radius, *a_xy_*, both at an intensity of 1/*e*^2^. If, in addition to diffusional motion, the fluctuations were caused by transitions between bright and dark states due to a photophysical process within the fluorophore such as a singlet-triplet intercrossing system, the fluorescence autocorrelation function would be affected and the kinetics of the relaxation processes would be accessible. In such cases, the interconversion time, *τ_i_*, can be incorporated into the autocorrelation equation by an exponential term as follows:

(2) $$G(t)=G_{D}(t)\left(1+\sum_{i}\gamma_{i}\cdot e^{-t/\tau_{i}}\right)$$

By fitting the diffusional part of the autocorrelation function, the diffusion coefficient, *D*, for the fluorescent species can be extracted via the following equation [55,56]:

(3) $$D=\frac{a_{xy}^{2}}{4\tau_{D}}$$

### 3.7. Convolution of the Autocorrelation Function with Oligomer Size Distributions

To extract the size distribution of the oligomers present in the soluble fraction of the aliquots, we convoluted a log-normal distribution of sizes with the full autocorrelation function. This convolution makes use of the following assumptions: (1) A log-normal distribution is the most probable distribution of oligomer sizes based on previous findings using single-molecule fluorescence spectroscopy [23,25] and kinetic molecular models [39,40]; and (2) the diffusion coefficient approximately scales with the cube root of the molecular weight [57]. The second assumption can be appropriately modified if one would try different models. For instance, the M^1/3^ scaling factor is adequate for globular proteins. In contrast, based on theoretical, hydrodynamic calculations on linear oligomers of tubulin, Krouglova *et al.* proposed a scaling factor of M^1/^*^n^*, with *n* ranging from 1.72 to 1.89 [58]. Nevertheless, there exist clear evidence that amyloidogenic aggregates are globular, at least in their early stages [59,60]. Therefore, we decided to use the M^1/3^ scaling factor.

The distribution of sizes (Equation 4), *P*(*x*), considering a total area of 1, is defined by *x_c_* and *σ*, the mean and standard deviation of the log-normal distribution, respectively.

(4) $$P(x)=\frac{1}{x\sqrt{2\pi \cdot \sigma^{2}}}\cdot e^{\frac{-{\left(\text{ln}\frac{x}{x_{c}}\right)}^{2}}{2\sigma^{2}}}$$

However, the equation for the distribution of the oligomer sizes must be discontinuous because the aggregation process involves the addition of discrete units. Therefore, the probability distribution in Equation 4 serves as weighting factors for the convolution with the overall autocorrelation function. Given that the overall diffusion coefficient (Figure 2) exhibits an approximately 50% decrease and that we are only analyzing the soluble oligomers, we considered sizes of up to twelve units. Once the excitation probe volume was calibrated, the overall equation for fitting the diffusional portion of the autocorrelation function using this convolution method (Equation 5) had the only adjustable parameters *x_c_*, *σ*, and *N*, the number of labeled molecules in the probe volume. This new method only increased the number of adjustable parameters in one, compared to the single diffusive species equation.

(5) $$G_{D,weighted}(t)=\sum_{x=1}^{12}\frac{P(x)}{\sum_{j=1}^{12}P(j)}\cdot G_{D}(t,\tau_{D,x})=\sum_{x=1}^{12}\frac{P(x)}{\sum_{j=1}^{12}P(j)}\cdot G_{D}(t,\frac{a_{xy}^{2}\cdot \sqrt[{3}]{x}}{4D_{mon}})$$

In Equation 5, the diffusion time associated with the species of size *x*, *τ_D,x_*, is given by Equation 6, assuming that the diffusion coefficient increases with size, in which *D_mon_* is the diffusion time of the monomer (250 μm^2^·s^−1^). In Equation 6, different models and escalation factors can be tried by substituting the index in the root, which in our case we have kept fixed as 3.

(6) $$\tau_{D,x}=\frac{a_{xy}^{2}\cdot \sqrt[{3}]{x}}{4D_{mon}}$$

The overall function for the fit of the experimental lifetime weighted autocorrelation functions includes the triplet dark state component as follows:

(7) $$G(t)=G_{D,weighted}(t)\left(1+\sum_{i}\gamma_{i}\cdot e^{-t/\tau_{i}}\right)$$

## 4. Conclusions

Using an advanced fluorescence correlation technique involving time-resolved fluorescence information, fluorescence lifetime correlation spectroscopy, we have studied the soluble oligomers on-pathway of amyloid fibrillization of a mutant SH3 domain. We have employed a dual-color technique with interleaved pulsed excitation and dual-channel detection that expands the application to two reporter fluorophores; therefore, two independent filtered autocorrelation functions can be obtained with a single measurement. The presence of the reporter dyes does not alter the amyloidogenic behavior of the protein because no appreciable differences were detected in the analyses performed from the AF488 and AF647 channels. We have developed a method to extract the oligomer size distributions through the convolution of the distribution probability with the diffusional autocorrelation equation. This method has revealed a rapid increase in oligomer size during the initial hours, which peaks at two days. After which, the oligomers continue growing and incorporate into the insoluble fraction. However, a population of soluble oligomers can still be detected after 9 days. This result confirms the dynamic equilibrium behavior of oligomers into mature fibrils and the fact that mature fibrils may be a source of small oligomers [25,61].

Our experiments also allow the determination of the kinetics of the monomer addition into oligomeric materials through two parallel methods via one measurement. The concentration of labeled molecules can be obtained from the number of molecules in the probe volume determined from the fits of the lifetime-filtered autocorrelation functions. In contrast, the single-molecule burst rates can be directly related to the concentration of the labeled monomers. These two independent methods provided comparable results. The rate of monomer loss for N47A Spc-SH3 exhibits a decay time between 1 and 5 h when incubated at a concentration of 1.1 mM, 37 °C, pH 3.2 and in the presence of 100 mM NaCl. Similar kinetics were observed using conventional ThT fluorescence measurements.

We have demonstrated the power of the dual-color PIE-FLCS method for obtaining accurate, relevant information regarding the amyloidogenesis process. The principal benefit of using a second color is to provide an independent, parallel reporter. However, this feature can be further exploited under different conditions. By increasing the concentration of the labeled monomers, the interaction between the monomers labeled with different dyes can be monitored using other techniques to provide additional insights into the oligomerization process—techniques such as cross-correlation [62] lifetime-filtered signals or single-molecule two-color coincidence detection [18,22]. A study of the intra-oligomer energy transfer between two fluorophores within the same aggregate can aid in obtaining a full description of the amyloid fibril formation mechanism. Research is currently being performed on these subjects.
